# A Conformationally Driven Mechanism in n‐Type Doping of Naphthalene Diimide‐Bithiophene Copolymer by 1H‐Benzimidazoles

**DOI:** 10.1002/advs.202402482

**Published:** 2025-02-20

**Authors:** Simone Cimò, Ilaria Denti, Lorenzo Rossi, Marco Cassinelli, Martina Rossi, Rossella Castagna, Garrett LeCroy, Alberto Salleo, Mario Caironi, Antonino Famulari, Chiara Castiglioni, Chiara Bertarelli

**Affiliations:** ^1^ Center for Nano Science and Technology Istituto Italiano di Tecnologia via Rubattino 81 Milano 20134 Italy; ^2^ Dipartimento di Chimica Materiali e Ingegneria Chimica Giulio Natta Politecnico di Milano Piazza Leonardo da Vinci 32 Milano 20133 Italy; ^3^ Department of Materials Science and Engineering Stanford University 476 Lomita Mall Stanford CA 94305 USA; ^4^ INSTM Consorzio Interuniversitario Nazionale per la Scienza e Tecnologia dei Materiali via Giuseppe Giusti 9 Firenze 50121 Italy

**Keywords:** benzimidazoles, conductivity, DFT modelling, doping, GIWAXS, P(NDI2OD‐T2)

## Abstract

N‐doped polymer semiconductors are of great interest in the field of organic thermoelectrics, as high‐conductive materials are still highly desired. In this framework, this paper aims to clarify whether the n‐doping of naphthalene diimide‐bithiophene copolymer, P(NDI2OD‐T2), by 1H‐benzimidazoles is a thermally activated process. The study interestingly demonstrates that a relevant change in conductivity, with an increase of more than three orders of magnitude with respect to pristine P(NDI2OD‐T2), occurs before the annealing process takes place, thus revealing that benzimidazole‐derived dopants are already active at room temperature. Moreover, despite the annealing time and temperature affecting the electrical conductivity of the system, their contribution is less relevant, with the increase of electrical conductivity limited to up to three times. The results from the electrical characterization of the samples are supported by infrared spectroscopy investigation and X‐ray analysis, revealing the marker bands of polaron and a manifest structural change between the undoped and the just‐doped P(NDI2OD‐T2) films, accompanied by only minor modifications during the annealing process. Finally, based on the results of density functional theory simulations, the conformational modifications of the 1H‐benzimidazole dopant molecules, induced by the interaction with the P(NDI2OD‐T2), are proposed as a possible mechanism explaining the effective doping at room temperature.

## Graphical abstract

This study shows that the n‐type doping of the P(NDI2OD‐T2) with 1H‐benzimidazoles is already effective at room temperature, leading to an increase in conductivity of more than three orders of magnitude before annealing. Spectroscopic and structural analyses, supported by DFT simulations, suggest that conformational changes in the dopant molecules upon intercalation within the polymer may account for the observed doping at room temperature.

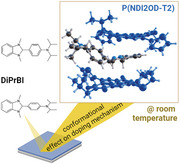

## 1 Introduction

The unique electrical properties, easy processability,^[^ ^1^ ^]^ and versatile chemical synthesis, have made π‐conjugated polymers promising materials to revolutionize many fields of low‐cost and flexible electronics.^[^ ^2^, ^3^ ^]^ At present, π‐conjugated small molecules and polymers find application in transistors,^[^ ^4^, ^5^, ^6^, ^7^ ^]^ thermoelectric devices,^[^ ^8^, ^9^, ^10^, ^11^, ^12^, ^13^, ^14^, ^15^, ^16^, ^17^, ^18^ ^]^ solar cells,^[^ ^6^, ^19^, ^20^ ^]^ light emitting diodes,^[^ ^21^, ^22^ ^]^ supercapacitors,^[^ ^23^ ^]^ sensors,^[^ ^24^, ^25^ ^]^ and neuromorphic devices.^[^ ^26^ ^]^ One of the key parameters of these materials is the conductivity of the organic semiconductor active layers. Doping an organic semiconductor either by reducing (*n*‐type doping) or by oxidizing (*p*‐type doping) can dramatically increase the charge carrier density and therefore the electrical conductivity,^[^ ^27^ ^]^ which is a fundamental requirement to develop efficient thermoelectric generators, and a technological need for nearly any efficient electronic device. Although for the development of thermoelectric devices both *p*‐type (hole conducting) and *n*‐type (electron conducting) materials are required, in the past the research was particularly focused on the study of the former.^[^ ^28^, ^29^, ^30^, ^31^, ^32^, ^33^ ^]^ The development of *n*‐type organic semiconductors has not been straightforward because of their difficult synthesis and because of their shallow lowest unoccupied molecular orbital (LUMO), which often leads to air instability.^[^ ^34^, ^35^, ^36^, ^37^, ^38^, ^39^ ^]^ In this framework, naphthalene diimide (NDI) – bithiophene (T2) copolymers exhibit relatively high electron mobility and rather good air stability,^[^ ^40^, ^41^, ^42^, ^43^ ^]^ which make them attractive for several applications.

Several studies have been conducted on this class of copolymers where both backbone^[^ ^31^, ^32^, ^33^, ^34^ ^]^ and side chains^[^ ^44^, ^45^, ^46^, ^47^ ^]^ have been systematically changed. Our work is focused on the widely used poly[N,N′‐bis(2‐octyldodecyl)‐naphthalene‐ 1,4,5,8‐bis(dicarboximide)‐2,6‐diyl]‐alt‐5,5′ ‐(2,2′ ‐dithiophene) P(NDI2OD‐T2), characterized by an electrical conductivity in its pristine state of ≈4 × 10^−7^ S cm^−1^.^[^ ^48^, ^49^, ^50^, ^51^, ^52^, ^53^, ^54^ ^]^ It is worth noting that the orientation of the chains changes upon the deposition method of the film. In particular, epitaxially grown films in different forms,^[^ ^55^ ^]^ form I and form II, as well as spin‐coated films were investigated through infrared spectroscopy together with an analytical model to individuate geometrical variables describing the polymer structure. Form I is distinguished by a segregated arrangement of NDI and T2 units, where NDI units are closely aligned on adjacent chains, as are T2 units. On the contrary in form II, a mixed stack of NDI and T2 units of the adjacent chain is present.^[^ ^49^ ^]^ Face‐on arrangement of the chains was proposed for form I, whereas the more thermodynamically stable form II, characteristic of high molecular weight samples, presents the polymer chain axis pointing out of the substrate. Spin‐coated films showed a T2 unit mostly parallel to the substrate^[^ ^54^, ^55^, ^56^, ^57^, ^58^ ^]^ in a face‐on conformation, with a dihedral angle of 38° between the NDI and the T2. Thermal treatment of pristine P(NDI2OD‐T2) affects its structure: in particular, melt annealing, followed by slow cooling, causes the transition to an edge‐on structure with the polymer segments running in a direction close to the perpendicular to the substrate.^[^ ^57^ ^]^

The *n*‐type doping of naphthalene diimide copolymers is hindered by the low stability of *n*‐type dopants in air. An electron donor should indeed have a highest occupied molecular orbital (HOMO) high enough to enable the transfer of electrons to the polymer LUMO.^[^ ^58^ ^]^ However, this characteristic makes the dopant rather unstable in air. A common approach to solve this issue is the use of air‐stable precursors, which can be converted in situ into reactive reducing agents.^[^ ^59^, ^60^ ^]^ Recently, 1H‐benzimidazoles have attracted great interest as *n*‐type dopants.^[^ ^61^, ^62^, ^63^ ^]^ P(NDI2OD‐T2) doped with the 4‐(1,3‐dimethyl‐2,3‐dihydro‐1H‐benzoimidazol‐2‐yl)‐N,N‐dimethylaniline (DMBI) or the 4‐(1,3‐phenyl‐2,3‐dihydro‐1H‐benzoimidazol‐2‐yl)‐N,N‐dimethylaniline (DPBI) shows an increase of electrical conductivity of ≈3 orders of magnitude with respect to the pristine state. However, at high dopant concentrations, phase segregation occurs, limiting the dopant efficiency.^[^ ^64^ ^]^ The N‐substitution of the aniline with longer linear or branched alkyl chains improves the dopant‐polymer miscibility, further increasing the electrical conductivity of the system. P(NDI2OD‐T2) doped with 4‐(1,3‐dimethyl‐2,3‐dihydro‐1H‐benzo[d]imidazol‐2‐yl)‐N,N‐diisopropylaniline (DiPrBI) exhibits electrical conductivity up to 7.2 × 10^−3^ S cm^−1^ recorded for doped P(NDI2OD‐T2).^[^ ^65^ ^]^ DMBI dimers have also been reported as effective *n*‐type dopants.^[^ ^66^ ^]^

Recently, X‐ray measurements highlighted how the face‐on orientation of the P(NDI2OD‐T2) crystallites is reduced upon doping.^[^ ^67^ ^]^ The remarkably higher conductivity in the direction parallel to the substrate plane, observed for doped P(NDI2OD‐T2), corroborated the idea of a change of morphology upon doping, with the π‐stacking direction from out‐of‐plane to in‐plane.^[^ ^67^ ^]^ Different doping mechanisms have been considered to explain the interaction and charge transfer between DMBI and P(NDI2OD‐T2). As previously reported, an acid‐base reaction with a hydride transfer from the dopant to the polymer was ruled out.^[^ ^68^ ^]^ Another interesting hypothesis involves the formation of an intermediate radical, singly occupied molecular orbital (SOMO), followed by a direct electron transfer from such molecular orbital to the LUMO of the polymer. This doping mechanism agrees with the traditional concept that benzimidazole derivatives are thermally activated upon annealing.^[^ ^69^, ^70^ ^]^ Despite some specific differences in the procedure to dope P(NDI2OD‐T2) with 1‐H benzimidazoles found in the literature,^[^ ^64^, ^66^, ^67^, ^68^, ^69^, ^70^, ^71^, ^72^ ^]^ this process often shares similar steps: the P(NDI2OD‐T2) solution containing the dopant is spin‐coated, and the deposited films are annealed under inert atmosphere for several hours. Annealing is often carried out at 150 °C for 6 h under N_2_ atmosphere,^[^ ^64^, ^65^, ^68^, ^81^ ^]^ but there are several examples in the literature with a lower annealing temperature (from 80 to 120 °C)^[^ ^45^, ^66^, ^67^, ^71^, ^72^, ^81^ ^]^ and from few minutes up to some hours.^[^ ^45^, ^71^, ^72^ ^]^

So far, thermal‐annealing was supposed to be required to activate the benzimidazole‐derived dopants and to promote a good packing of the polymer chains, as for most of the conjugated polymers in the pristine state. In order to reach the optimal process condition for doping, we systematically changed the annealing temperature and time. Here, we show that a strong change of conductivity is achieved before the thermal post‐processing, which allows just for a less significant further increase, demonstrating that the 1H‐benzimidazoles do not require thermal activation above room temperature for the charge transfer to occur. A molecular modeling study, based on density functional theory (DFT), suggests a new model for the doping mechanism providing a rationalization of the experimental outcomes.

## 2 Results and Discussion

To study the effect of annealing temperature and time on the *n*‐doping process, we selected the well‐studied P(NDI2OD‐T2) and the 4‐(1,3‐dimethyl‐2,3‐dihydro‐1H‐benzo[d]imidazol‐2‐yl)‐N,N‐diisopropylaniline (DiPrBI) as dopant, given the high conductivities reported for this pair.^[^ ^65^ ^]^ Films of P(NDI2OD‐T2) at two different concentrations of the dopant were prepared, namely, 76%_(mol/mol)_(20 wt%), corresponding to the optimal dopant concentration according to the previous results,^[^ ^65^ ^]^ and at 150%_(mol/mol)_ (33 wt%) possibly giving rise to dopant segregation. Dopant concentration is expressed as the ratio between dopant moles and the number of P(NDI2OD‐T2) repeating units.

**Figure 1:**
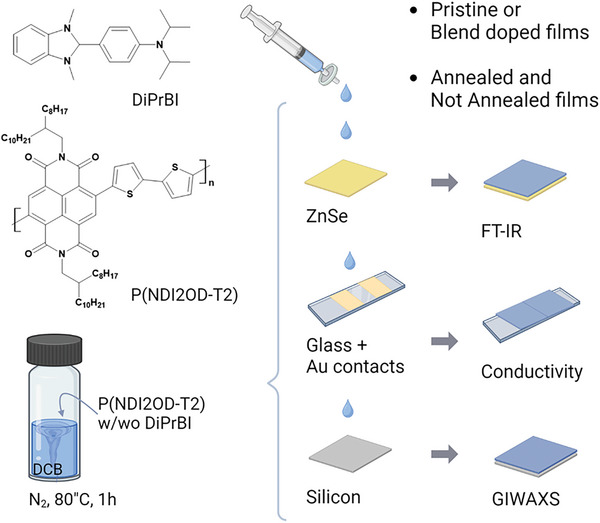
The chemical components (DiPrBI dopant and P(NDI2OD‐T2) polymer) and the experimental procedure followed for the sample preparation. Pristine or doped films are deposited on different substrates suitable for the different experimental characterizations. Films are characterized as deposited or after a thermal annealing.

The electrical, spectroscopical, and morphological properties of both pristine and doped P(NDI2OD‐2) were evaluated (**Figure** **1**). Details regarding sample preparation and measurements are reported in the Experimental Section.

### 2.1 Electrical Properties

**Figure 2:**
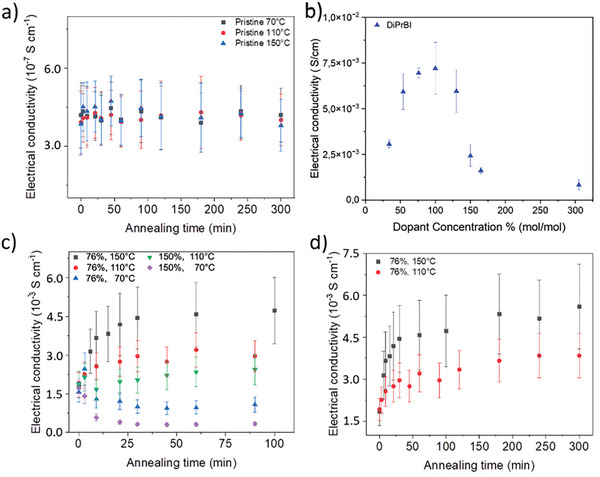
A) electrical conductivity of films of pristine P(NDI2OD‐T2) annealed at 70, 110 and 150 °C over time. B) electrical conductivity of a P(NDI2OD‐T2) film doped at different concentrations with DiPrBI after 5h annealing at 150 °C. C): electrical conductivity of DiPrBI‐doped P(NDI2OD‐T2) spin‐coated films annealed at different temperatures over time. Black, red, and blue dots refer to films of P(NDI2OD‐T2) 76% doped with DiPrBI annealed at 150, 110, and 70 °C, respectively. Green and violet dots refer to films of P(NDI2OD‐T2) 150% doped with DiPrBI annealed at 110 and 70 °C, respectively. D): electrical conductivity of P(NDI2OD‐T2) 76% doped with DiPrBI annealed for a longer period (up to 5h).

To provide a benchmark for doped samples, the conductivity of pristine P(NDI2OD‐T2) films annealed at 70, 110, and 150 °C were first measured (**Figure** **2**A). Annealing temperatures have been chosen taking into account the range of values reported in the literature, which spans from very mild conditions up to 150 °C.^[^ ^66^, ^67^, ^68^, ^69^, ^70^, ^71^, ^72^ ^]^ For all the samples of the pristine polymer, the electrical conductivity remains stable ≈4.0 × 10^−7^ S cm^−1^, irrespective of annealing time and temperature. This result is in agreement with the literature, reporting significant changes of P(NDI2OD‐T2) morphology,^[^ ^52^, ^57^ ^]^ hence of electrical conductivity, only at very high temperatures (>300 °C) or annealing assisted by rubbing.

Concerning the doping ratio, we analyzed samples at the optimal DiPrBI dopant concentration of 76%_(mol/mol)_, according to the previous results,^[^ ^65^ ^]^ and at a higher value (150%_(mol/mol)_) where the supposed phase segregation occurs (Figure 2B), leading to a worsening of the electrical conductivity.

The most interesting finding is the conductivity of the as‐spun doped films: without any annealing process, the electrical conductivity reaches values of the order of 10^−3^ S cm^−1^, which is almost four orders of magnitude higher than values of the non‐doped polymer. Moreover, all samples demonstrate similar electrical conductivity at ≈1.8 × 10^−3^ S cm^−1^, independently from the specific dopant concentration. These experimental results demonstrate that the 1H‐benzimidazole is already active at room temperature, without annealing, and a thermally‐activated doping mechanism is not required.

Worth noting that a further change in conductivity is obtained once the DiPrBI:P(NDI2OD‐T2) films are annealed, and here different results are obtained depending on both the dopant concentration and the annealing temperature. First, at both annealing temperatures of 70 and 110 °C, a too high concentration of dopant (i.e., molar dopant ratio 150%) leads to lower electrical conductivity with respect to the one measured in the case of the 76% DiPrBI:P(NDI2OD‐T2) molar ratio, regardless of annealing time (Figure 2C). This confirms the conclusions reported by Chabinyc et al.^[^ ^64^ ^]^ and Bertarelli et al.^[^ ^65^ ^]^ about the worsening of the doping efficiency at high dopant concentrations, ascribed to phase segregation phenomena.

For annealing performed at higher temperatures (i.e., 110 and 150 °C), the electrical conductivity *σ* of the 76% doped P(NDI2OD‐T2) samples over annealing time up to 5 h follows an interesting trend (Figure 2D): an almost constant increase in the first 15–30 min, then reaching a plateau for a longer annealing time. Accordingly, we speculate that the final structure of the doped material (i.e., degree of doping and morphology of the doped phase) is reached through different stages. The first occurs at room temperature, and it is responsible for a dramatic increase (>5000 times) in electrical conductivity. The second one is triggered by the annealing process; it occurs within the first 15–30 min of annealing and it causes an increase of electrical conductivity of ≈50–100%. Finally, the last stage is slower; it requires up to 4–5 h causing a further increase of electrical conductivity of ≈20–30%. Overall, the total contribution of the annealing process to the thin films electrical conductivity amounts to an increase of ≈2–3 times only.

Surprisingly, annealing at the lowest temperature (70 °C) seems to be detrimental (Figure 2C), showing a decreasing conductivity value as a function of different annealing times. This suggests that competitive mechanisms are responsible for the conductivity changes with thermal post‐process, which is ruled by the annealing temperature.

#### 2.1.1 Spectroscopic Characterization

Infrared spectroscopy (FT‐IR) on P(NDI2OD‐T2) doped film with DiPrBi (20 wt%, 76% mol mol^−1^) provides independent proof that the doping takes place already at ambient temperature. Films were dropping cast on the Zinc Selenide window of a temperature‐controlled cell, which was sealed in a nitrogen environment. One sample was tested at room temperature, without any annealing treatment, and a second sample was annealed at 110 °C. Spectra were acquired for 120 and 145 min, respectively. During all the experiments exposure to oxygen was prevented.

**Figure 3:**
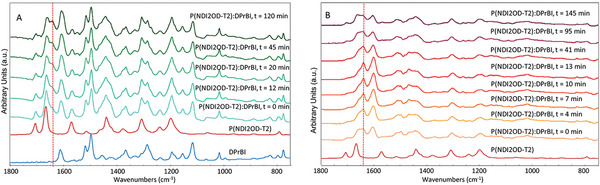
A) FT–IR spectra of a film cast from a solution of P(NDI2OD‐T2)/ DiPrBI in dichloromethane. The sample was monitored for 120 min to show the time‐dependent evolution of doping, without annealing. The spectra of the dopant molecule and the pristine P(NDI2OD‐T2) correspond to the bottom blue line and the red line, respectively. B) Evolution over the annealing time of the FT–IR spectrum of a sample of P(NDI2OD‐T2)/DiPrBI, heated at 110 °C. The feature at 1638 cm^−1^ is a marker band associated with the polaron^[^ ^68^ ^]^ and is highlighted by a vertical red broken line.

The infrared measurements show that, already at room temperature (**Figure** **3**A) the vibrational bands assigned to infrared active normal modes localized on the charged defect, namely on the polaron, are present. Indeed, the main marker band of the polaron at 1638 cm^−1^,^[^ ^68^ ^]^ highlighted by a broken line in Figure 3A, is detectable as a pronounced shoulder, already at t = 0 and grows with time, thus indicating that the polarons forms already at ambient temperature, as was suggested by the remarkable conductivity increase of the unannealed doped samples.

Interestingly Figure 3B shows that the annealing accelerates the appearance of the polarons features, which are strong as soon as the annealing temperature is reached (t = 0). However, at the longest times the spectra of Figures 3A,B, show that a comparable polaron concentration is reached, for both the unannealed and the annealed samples. The above findings were unexpected based on a previous study on the vibrational response of doped P(NDI2OD‐T2).^[^ ^68^ ^]^ In Denti et al.,^[^ ^68^ ^]^ films of DiPrBI‐doped P(NDI2OD‐T2) were prepared by casting from a chloroform solution and analyzed via FT‐IR spectroscopy.

In this case, polaron vibrational bands did not show when spectra were recorded s on the deposited sample, while the marker bands of the polaron at 1638 cm^−1^ – together with its other characteristic vibrational features – appeared only after the annealing of the film. It is reasonable that such a different behavior is ascribed to the remarkable impact of the solvent on the film morphology and the growth of doped polymer domains. The high volatility of chloroform accelerates the solidification process and probably prevents the formation of the most stable doped form, which is obtained in a second step when the annealing promotes the doping process, which is limited by its rather slow kinetics.

### 2.2 Film Morphology

**Figure 4:**
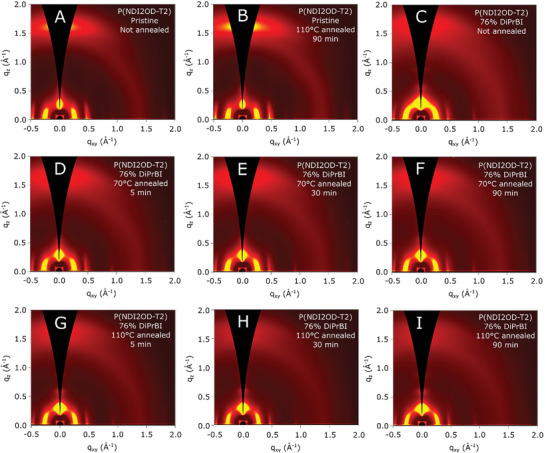
GIWAXS data for A) not annealed pristine P(NDI2OD‐T2); B) pristine P(NDI2OD‐T2) annealed at 110 °C for 90 min; C) not annealed 76% DiPrBI doped P(NDI2OD‐T2); D–F) 76% DiPrBI doped P(NDI2OD‐T2) annealed at 70 °C for 5, 30 and 90 min, respectively; G–I) 76% DiPrBI doped P(NDI2OD‐T2) annealed at 110 °C for 5, 30 and 90 min, respectively.

**Figure 5:**
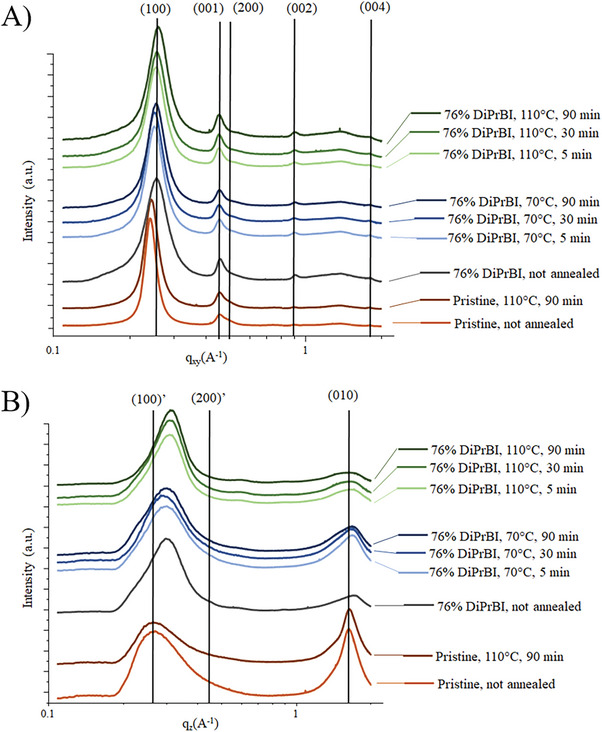
A). Vertical line‐cuts (in plane direction) and B). horizontal line‐cuts (out of plane direction) of the 2D GIWAXS spectra for P(NDI2OD‐T2) doped with DiPrBi, 76%. Both graphs are shown from bottom to top: pristine samples not annealed and annealed at 110 °C for 90 min (brown lines); doped not annealed sample (grey line); doped samples annealed at 70 °C for 5, 30, and 90 min (blue line); doped samples annealed at 110 °C for 5, 30 and 90 min (green line).

To study the effects of the different annealing processes on the thin film structure and crystallinity, Grazing Incident Wide Angle X‐ray Scattering (GIWAXS) measurements were performed on samples subjected to different annealing temperatures and times. Representative GIWAXS patterns for all the samples are shown in **Figure** **4**. The reduced 1D line profiles reported in **Figure** **5** are taken along the horizontal (in the plane, *q_xy_*) and approximately vertical (out of the plane, ≈*q_z_*) scattering directions. The out‐of‐plane (≈*q_z_*) scattering peak at q ≈1.6 Å^−1^ is attributed to the (010) planes along the π‐stacking direction. There are a number of scattering peaks along the in‐plane (*q_xy_*) scattering direction attributed to the (001) and (100) planes corresponding to periodicity along the backbone repeat units and in‐plane lamellar stacking directions. Figure 5 highlights that up to four orders of lamellar stacking and up to two orders of backbone repeat unit scattering peaks can be observed in the in‐plane direction, agreeing with previous GIWAXS studies that showed P(NDI2OD‐T2) has exceptional in‐plane ordering.^[^ ^57^ ^]^

To evaluate annealing effects on pristine P(NDI2OD‐T2), we performed GIWAXS measurements in an as‐deposited state and after annealing at 110 °C for 90 min.

Pristine P(NDI2OD‐T2) exhibits a face‐on orientation (Figure S1, Supporting Information) as seen in the literature.^[^ ^52^, ^57^ ^]^ The sample annealed well below the melting temperature also displays a largely face‐on structure with little changes to lamellar or π‐stacking spacings (Figures 4A,B). Interestingly, the annealed sample displays an overall increase in crystallinity as measured from lamellar scattering pole figures (Figures S1, S2, Supporting Information), with an increase in both the edge‐on crystallite scattering contribution and a greater increase in the face‐on crystallite scattering (Figure S1A, Supporting Information) that results in a greater face‐on crystallite texture. Nevertheless, these structural changes between pristine and annealed films do not correlate with a significant change in thin‐film conductivity (Figure 2A).

Conversely, the unannealed 76% doped sample exhibits a dramatic change in its morphology; particularly, the *q_z_* (010) π‐stacking scattering peak (q ≈1.6 Å^−1^) decreases in intensity and broadens significantly in the doped sample, and the *q_xy_* (100) lamellar scattering peak (q ≈0.3 Å^−1^) shifts to higher *q*‐values. The broadening of the *q_z_* (010) peak indicates that the dopant intercalation alters the order of the π‐stacking.^[^ ^65^, ^66^, ^67^ ^]^ In agreement with,^[^ ^65^ ^]^ a possible explanation of this behavior is that the dopants intercalate between the NDI units, introducing disorder in the π‐stacking distances. This interpretation suggested the geometry we adopted for the set‐up of the molecular model mimicking the P(NDI2OD‐T2)–dopant complex, which will be discussed in Section 2.3. A comparison between the 70 and 110 °C in‐plane data shows that the (100) lamellar peak (Figure 5a) moves toward higher *q_xy_* values with increasing annealing temperature and time. In particular, the (100) peak of the 70 °C, 90 min annealed sample has the same *q* value as the sample annealed at 110 °C for 5 min. Additionally, increasing annealing temperature and time generally leads to increases in the face‐on texture of the thin films and increases in the relative degree of crystallinity (RDoC), with more pronounced increases in RDoC for the 110 °C annealed samples (Figure S2, Supporting Information).

The (001) backbone peak does not change in position or intensity/shape, confirming that the doping process does not influence the intramolecular repeat distance along the polymer chain axis as well as the intermolecular order that must be present to give rise to the backbone peaks.

Both the 110 and 70 °C samples exhibit a decrease in the coherence length and intensity of the *q_z_* (010) peak concerning the *q_z_* (100)’ peak. In particular, this behavior is more pronounced in the 110 °C series. Furthermore, a shoulder ≈1.6 Å^−1^ arises in the 70 °C, 90 min annealed sample and in all samples annealed at 110 °C. These changes are consistent with a perturbation of the π‐stacking direction, characterized by a shorter‐range order with respect to the pristine case, as previously discussed.

The *q_z_* (200)’ peak gains intensity in the 110 °C series (Figure 5A; Figure S1C, Supporting Information), though the in‐plane lamellar scattering peaks also gain in intensity resulting in an overall increase in the RDoC of the film as measured from lamellar scattering. Moreover, the broadening of the peak assigned to π–stacking (i.e., the intermolecular transport direction) upon doping, indicates a higher disorder within the crystallites of the doped samples than in the pristine samples. This demonstrates that the dopant might be intercalated in the polymer stacks, causing the chains to rearrange, and possibly locally increase the π‐stacking distance. GIWAXS lineout data (Figure 5) and lamellar pole figure analysis (Figures S1, S2, Supporting Information) show that the 110 °C annealed and doped series displays a higher RDoC and higher relative fraction of face‐on crystallites. Moreover, the observed morphology is strongly correlated with the electrical conductivity data, where higher electrical conductivity is found for the 110 °C series (Figure 2c). It is reasonable to conclude that the increase in overall thin film crystallinity and the increase in face‐on texture provide an effective microstructure that enhances charge transport.

### 2.3 Molecular Modeling Results

**Figure 6:**
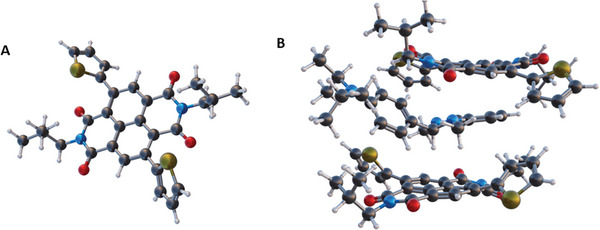
A) A view of T‐NDI2Bu‐T. B) A view of the model system composed of DiPrBI and two T‐NDI2Bu‐T units resulting from DFT‐D geometry optimizations.

While a hydride transfer reaction has been ruled out,^[^ ^68^ ^]^ an electron transfer between the dopants radical SOMO and the polymer LUMO is a plausible hypothesis reported in the literature to explain the doping mechanism of benzimidazole derivatives for P(NDI2OD‐T2).^[^ ^69^ ^]^ The energetical justification for this doping mechanism is based on the radical formation during the annealing process, implying that the dopants are thermally activated. However, this hypothesis is in contrast with our experimental data because we observe a substantial increase in the electrical conductivity before thermal annealing is performed, indicating that the dopant molecules are already active at room temperature. In addition, GIWAXS analysis suggests that the alteration of the *q_z_* (010) peak, associated with the order, i.e., coherence length, along the π‐stacking direction, might be caused by the presence of dispersed dopant molecules between the polymer units. According to these considerations, we investigated an alternative mechanism in which the dopant electrons could be activated by intermolecular interactions with the polymer at room temperature. When molecular modeling approaches are employed, two main points should be carefully addressed in the simulation of molecular processes to get reasonable insights from the results: 1) the level of the theory^[^ ^73^ ^]^ and 2) the model system involved in the simulations.^[^ ^74^ ^]^ Specifically, we hypothesize that the dopant could modify its structure (i.e., its conformation) while intercalating between the narrow polymer lamellae and that such structural modification is accompanied by a perturbation of the dopant electronic structure. Therefore, after dopant intercalation, new electronic transitions might be accessible to the dopant electrons, thus allowing for an electron transfer to the polymer. As a first step of our computational study, we performed DFT‐D (i.e., DFT plus Grimme empirical corrections, see experimental) calculations aiming to evaluate the amount of energy involved in the interaction between the dopant molecules and the polymer. To maintain good accuracy together with a reasonable computational cost, we performed a geometry optimization on a simplified model system composed of a single DiPrBI molecule interacting with two repeating units of P(NDI2OD‐T2). Moreover, to further reduce computational costs, the long‐branched alkyl chains on the NDI units have been replaced with isobutyl side groups (T‐NDI2Bu‐T, **Figure** **6**A). The results of this optimization (Figure 6B) clearly show a favorable interaction between the NDI units of T‐NDI2Bu‐T and the benzimidazole part of DiPrBI, whereas the isopropyl chains of DiPrBI orient themselves toward the alkyl chains of the polymer units. The corresponding interaction energy is evaluated as the usual standard supermolecule approach:

(1) $$E_{\left(\mathrm{interaction}\right)}=E_{\left(\mathrm{SYSTEM}\right)}-E_{\left(\mathrm{DiPrBI}\right)}-2E_{\left(T-\mathrm{NDI}2\mathrm{Bu}-T\right)}$$

where E _(SYSTEM)_ is the total energy of the system composed of DiPrBI sandwiched between two T‐NDI2Bu‐T units; E _(DiPrBI)_ is the total energy of DiPrBI in its absolute conformational minimum and E _(T‐NDI2Bu‐T)_ is the total energy of a T‐NDI2Bu‐T unit. The results show a quite high interaction energy of ≈−56 kcal mol^−1^. It is clear, by definition, that the negative sign indicates that the interaction – following the change transfer – between the moieties stabilizes the system. Taking this as a starting point, we can make a further simple hypothesis: the stabilization reached by this interaction can cause conformational changes (i.e., distortion from its absolute minimum) to the dopant molecules, thus activating and promoting electron transfer to the polymer.

**Figure 7:**
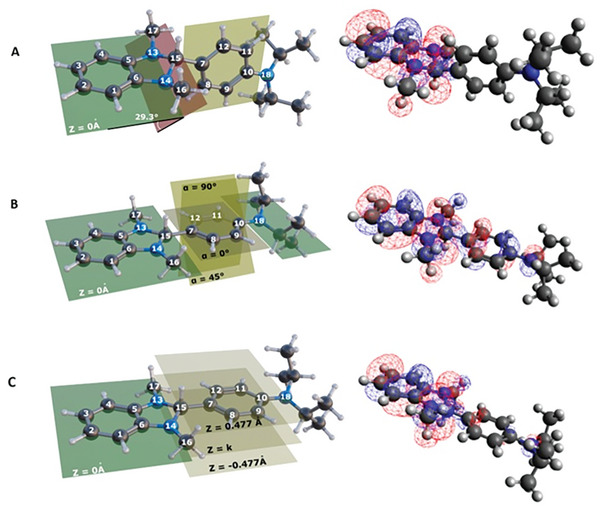
A) Spatial representation of DiPrBI structure in the absolute minimum. B) First set of guess geometries, C) Second set of guess geometries.

As previously stated, we hypothesize a doping mechanism involving significant geometrical modifications of the dopant, in analogy to what was described previously in literature for this class of compounds.^[^ ^81^, ^82^ ^]^ Specifically, given the marked planar structure of the NDI units of the polymer, we assume a conformational modification, which we can describe as a quasi‐planarization of the dopant structure while intercalating within the polymer lamellae. To assess the feasibility of this distortion, we performed a systematic study by designing a set of plausibly involved DiPrBI geometries, obviously assuming that some of these structures can be sampled by the intercalation of the dopants within the polymer lamellae. The DiPrBI molecule in its absolute minimum conformation is reported in **Figure** **7**A. In particular, the atoms 5, 6, 13, and 14 define the *xy* plane (z = 0 Å), and the origin of the cartesian axis is placed in the middle point of the segment connecting atoms 13 and 14. The dihedral angle between the *xy* plane and the plane passing through atoms 13, 14, and 15 is 29.3°. The dihedral angle between the plane passing through atoms from 7 to 12 and the *xy* plane is 72°. The corresponding calculated HOMO energy level for this conformation is between −4.40 eV (in vacuum) and −4.70 eV (considering dichloromethane as an implicit solvent in the model). The level of the theory has been chosen to ensure both the reproducibility of cyclic voltammetry (CV) experimental values and reasonable computational costs. The experimental HOMO energy level obtained by CV is −4.59 eV.

We designed two sets of geometries. In the first one, the atoms 15 and 18 are constrained to lay on the *xy* plane (*z* = 0 Å) and the dihedral angle is systematically scanned between the two phenyl groups (Figure 7B) while performing geometry optimizations of the remaining internal degrees of freedom. The HOMO energy changes upon the molecule distortion have been evaluated together with the energy cost with respect to the DiPrBI absolute minimum conformation, and the results are reported in **Table** **1**. To design the second set of geometries we instead forced atoms 7, 8, 9, 10, 11, 12, and 15 on the same plane parallel to the *xy* plane (*z* = 0 Å) and placed at different heights (*z* = *k*). We then systematically varied the distance *k* between the two planes (Figure 7C). The calculation results are reported in **Table** **2**.

**Table 1:** Calculated HOMO energy (eV) upon molecule dihedral angle rotation and energy cost with respect to the DiPrBI absolute minimum conformation (see Figure 7).

Dihedral Angle [°]	HOMO [eV]	Cost [kcal mol^−1^]
0	−3.5	47
30	−3.8	44
45	−3.7	44
60	−3.7	48
90	−4.0	41

**Table 2:** Calculated HOMO energy (eV) upon molecule distortion and energy cost with respect to the DiPrBI absolute minimum conformation. Z is the distance between the plane defined by the phenyl ring composed of carbon atoms 1–6 and the distance between the parallel plane passing through carbon atoms 7–12 (see Figure 7).

Z [Å]	HOMO [eV]	Cost [kcal mol^−1^]
−0.477	−3.3	105
−0.239	−3.3	84
0.000	−3.5	64
0.239	−3.8	44
0.477	−4.1	30

The calculations show that these geometrical distortions cause a substantial rise of the HOMO energies of the dopant, toward matching the LUMO of the polymer, possibly reaching values even higher than the polymer LUMO. Furthermore, the energetic cost for the corresponding geometrical distortion is comparable to the interaction energy involved in the intercalation step of the doping process, meaning that these interactions can compensate for the energy penalty.

Moreover, we calculated the energy of the homolytic or heterolytic C─H bond breaking as the difference between the energy of products and reagents (i.e., of R‐H →  R^•^ + H ^•^ and R‐H →  R^+^ + H^−^). The calculation was carried out by DFT using the B3LYP/6‐31G^**^ approach. In the first case, we obtain the energy required to remove a hydrogen atom from the DiPrBI molecule which results in 83.40 kcal mol^−1^. In the second case, the energy required to remove a hydride from the DiPrBI molecule is in the order of 478 kcal mol^−1^, indicating a less favorable process.

To summarize, the model here proposed highlights the role of the polymer‐dopant interactions in promoting the charge (electron) transfer from the HOMO of the dopants to the LUMO of the polymer (−4.0 eV). According to our calculations, there exist perturbed conformations – likely originating from the intercalation processes – that would substantially reduce or eliminate the energy barrier for the charge carrier injection. This charge transfer model suggests a doping mechanism that can explain why benzimidazole derivatives are already active at room temperature, before annealing.

## 3 Conclusions

A collection of experimental evidence interestingly demonstrates that n‐type doping of P(NDI2OD‐T2) by 1H‐benzimidazoles is already active at room temperature. We show that before the thermal treatment takes place, the electrical conductivity of P(NDI2OD‐T2):DiPrBI thin films already increases by more than three orders of magnitude with respect to the pristine polymer. This is a clear indication that DiPrBI is already active at room temperature, whereas annealing has only a minor effect, causing a further two‐three time increasing of electrical conductivity values. The increase of conductivity upon annealing seems to occur in two steps, at shorter and at longer times. First, it can raise the number of doping‐induced charge carriers, by providing the energy supply necessary for the intercalation of some dopant molecules, which, in the sample as deposited, did not reach the suitable configuration for charge injection. Moreover, as demonstrated by GIWAXS experiments, it can promote a refinement of the doped film morphology, which exploits the interchain hopping mechanism between NDI units and thereby increases conductivity. GIWAXS measurements reveal that the morphology of the doped film is remarkably different from that of the non‐doped polymer film, characterized by structural disorder (distribution of π‐stacking distances and orientational disorder), which is compatible with dopant intercalation between NDI units. Moreover, the GIWAXS analysis suggests that the annealing at 110 °C results in increasing the RDoC and the face‐on texture, while the annealing at lower temperature (70 °C) only slightly increases the number of face‐on domains. This increase in crystallinity and face‐on texture provides a favorable microstructure that correlates with the conductivity trends observed for films annealed at such temperatures.

To rationalize the experimental findings, we carried out a thorough theoretical study based on Quantum Chemical modeling focusing on the relationship between the molecular and electronic structure of the dopant. This study shows the importance of polymer‐dopant interactions in the doping mechanism. We demonstrate that, due to a quasi‐planarization, the HOMO of the dopant rises significantly thus reducing or eliminating the barrier of the electron injection into the polymer LUMO orbital. The planarization of the dopant molecule is expected to occur during intercalation processes, because of the interaction with NDI units: the energy cost is compensated by the large intermolecular energies. According to the calculations, we demonstrated that conformations accessible during intercalation exist, which would promote the charge transfer. This simple model, proposed as a new doping mechanism, complements the currently accepted processes, which cannot be entirely discarded due to the chemical nature of the dopant used, which likely explains why DiPrBI is already active at room temperature. Overall, the generation of the charge carrier and the predominance of face‐on configuration of the doped polymer in the as‐deposited films indicates that the doping P(NDI2OD‐T2) by 1H‐benzimidazoles is a non‐thermally activated mechanism. This study highlights the relevance of solid‐state intermolecular interactions between dopant and polymer, leading to the doping mechanism. This paves the way for the development of strategies to design new stable organic dopants, activated by structural relaxation.

## 4 Experimental Section

### Dopant Synthesis

All chemicals were purchased from Sigma–Aldrich, TCI Chemicals, or Fluorochem, and they were used as received. Chemicals, intermediates, and products were stored in the fridge, inside a sealed flask containing Argon. P(NDI2OD‐T2) (Polyera ActivInk N2200, Mn = 150 kDa) was purchased from Ossila Lim. DiPrBI was synthesized as previously reported.^[^ ^65^ ^]^

### Dopant Synthesis—Cyclic Voltammetry

The electrochemical behavior of DiPrBI was studied by cyclic voltammetry (CV), at a potential scan of 0.05 Vs^−1^, in a 0.5 mm dichloromethane solution. The solution was purged with nitrogen before each experiment. 0.1 m tetrabutylammonium perchlorate (TBAP) was used as the supporting electrolyte. The experiments were carried out using a PGSTAT 302N potentiostat. The working electrode was glassy carbon (GC), and the counter electrode was a platinum wire. The operating reference electrode was an aqueous saturated calomel one (SCE). All measured potentials were referred to as the Fc^+^/Fc couple whose potential was measured in the same working conditions as the tested materials. The oxidation potentials of the passivation materials were determined by measuring the onset of the baseline and the tangent to the oxidation peak. The HOMO level was calculated with ferrocene (Fc^+^/Fc) as a reference redox couple using the following equation:

(2) $$E_{\mathrm{HOMO}/\mathrm{LUMO}}=\left[-\left(E_{\mathrm{onset}}-E_{\mathrm{onset}}\left({\mathrm{Fc}}^{+}/\mathrm{Fc}\right)\right)\right]-4.8\mathrm{eV}$$

### Electrical Characterization

### Electrical Characterization—Contact deposition

Low‐alkali 1737F Corning glass was used as substrate. First, the glasses were cleaned through an ultrasonic bath of Milli‐Q water, acetone, and isopropyl alcohol for 10 min for each step, and then exposed to O_2_ plasma at 100 W for 10 min. Subsequently, the electrodes were thermally evaporated using a shadow mask, obtaining electrical contact lines made of 1.5 nm thick Cr adhesion layer and 25 nm thick Au film, 2 mm wide, long between 1 and 1.5 cm and distant to each other 8 mm.

### Electrical Characterization—Film Deposition

Polymer film preparation was carried inside a glovebox in an N_2_ atmosphere. P(NDI2OD‐T2) (Polyera ActivInk N2200, purchased from Ossila Lim, Mn = 150 kDa,) was dissolved in 1,2‐diclorobenzene (DCB, concentration of 10mg mL^−1^). The resulting solution was stirred at 80 °C for 1h. The mixture was loaded into a syringe and filtered using a 0.45 µm pore size polytetrafluoroethylene filter. Aliquots of 50 µL of such solution were added to vials containing 0.124 and 0.245 mg of DiPrBI, enough to dope 50 µL of the solution at 76%_(mol/mol)_ (20 wt%) and 150% _(mol/mol)_ (33 wt%) respectively. All concentrations were expressed as dopant moles divided by P(NDI2OD‐T2) repeating unit moles. Glass substrates were cleaned with isopropyl alcohol and acetone, then treated with an oxygen plasma for 15 min, and quickly introduced inside the glovebox. P(NDI2OD‐T2) films were spin‐coated at 1000 rpm for 60 s and then at 3000 rpm for 10 s. For each dopant concentration, one sample was measured without thermal treatment, the remaining were annealed inside a glovebox in an N_2_ atmosphere on a hot plate.

### Electrical Characterization—Electrical Conductivity

The electrical characterizations were carried out at room temperature in an N_2_ atmosphere using a Wentworth Laboratories probe station with a semiconductor device analyzer (Agilent B1550A). Then, the electrical conductivity was calculated through the linear fit of the I–V characteristic curves and the sample's geometries. In particular, to measure the thickness of the thin films, an alpha‐step IQ profilometer from KLATencor was employed. The samples were scratched with a toothpick in multiple regions and the thickness of the films was measured in different points of the sample. This procedure was necessary to assess the uniformity of the conductive layer. The samples were between 30 and 50 nm thick.

### Spectroscopic Characterization‐Infrared Spectroscopy

Because of the difficulty in the acquisition of spectra with a good signal‐to‐noise ratio on thin films, FT–IR spectra were acquired on drop‐cast films. A solution of P(NDI2OD‐T2) doped with DiPrBI was prepared as described in Experimental Section under sub head “Film Deposition” and drop‐casted on a zinc selenide window of the heating cell (Linkam heating cell, FT‐IR 600) operating in a nitrogen atmosphere. Films were drop cast in air, then the cell was quickly sealed, and the solvent was let to evaporate in a nitrogen environment. A temperature‐control stage was used to perform the measurements. The cell was mounted on a Thermo Nicolet NEXUS FT–IR spectrometer (4 cm^−1^ resolution, 128 scans) equipped with a ThermoElectro Continuµm FT–IR Microscope (4 cm^−1^ resolution, 128 scans). The spectra were recorded in transmission mode, for 120 min, keeping the sample at ambient temperature and avoiding exposure to oxygen. In addition, the evolution of the IR spectrum with the annealing time was followed for a sample cast in the same way and heated at 110 °C with a fast‐heating ramp (50 °C min^−1^) to reach the desired temperature.

### X‐Ray Characterization

### X‐Ray Characterization—Film Deposition

Native oxide p+ silicon substrates 2 cm^2^ substrates were cleaned with acetone, isopropyl alcohol, and ethanol. The film preparation was conducted in an N_2_ glovebox (<1 ppm of O_2_) as previously described, using 890 µL of polymer solution for each sample. One sample was measured without thermal treatment, the others were annealed for 5, 30, and 90 min at 110 and 70 °C.

### X‐Ray Characterization—GIWAXS

Grazing‐incident wide‐angle X‐ray scattering (GIWAXS) measurements were conducted at the Stanford Synchrotron Radiation Lightsource (SSRL) on beamline 11–3 equipped with an area detector (Rayonix MAR‐225), using an incident energy of 12.73 keV. The measurements were performed in a chamber provided a helium flux, to minimize air scattering and beam damage to the sample. The sample and detector distance (321 mm) was calibrated through a LaB6 polycrystalline standard and the incident angle was chosen at 0.1°. Data analysis was performed with the Igor Pro software packages Nika 1D SAXS29^[^ ^75^ ^]^ and WAXStools.^[^ ^76^ ^]^ The data were normalized by detector counts and thickness (C1).

### Molecular Modeling

All the calculations (geometry optimizations, PES investigation, and HOMO and LUMO energies) were performed using the Orca package,^[^ ^77^ ^]^ by employing the B3LYP functional^[^ ^78^ ^]^ (including explicit Grimme empirical van der Waals contributions^[^ ^79^ ^]^) together with the standard 6–31G^**^ basis set. The geometries were instead drawn and analyzed using Avogadro.^[^ ^80^ ^]^

## Conflict of Interest

The authors declare no conflict of interest.

## Supporting information



Supporting Information

## Data Availability

The data that support the findings of this study are available from the corresponding author upon reasonable request.
