# Supplementary material for: A Conformationally Driven Mechanism in n‐Type Doping of Naphthalene Diimide‐Bithiophene Copolymer by 1H‐Benzimidazoles
Source: Adv Sci (Weinh). 2025 Feb 20;12(15):2402482. doi: 10.1002/advs.202402482 (PMC12005812; doi:10.1002/advs.202402482)
Supplement: Supplementary file 1 — Supporting Information [file ADVS-12-2402482-s001.pdf]

ADVANCED SCIENCE
Open Access

Supporting Information

for *Adv. Sci.*, DOI 10.1002/advs.202402482

A Conformationally Driven Mechanism in n-Type Doping of Naphthalene Diimide-Bithiophene Copolymer by 1H-Benzimidazoles

*Simone Cimò, Ilaria Denti, Lorenzo Rossi, Marco Cassinelli, Martina Rossi, Rossella Castagna, Garrett LeCroy, Alberto Salleo, Mario Caironi, Antonino Famulari, Chiara Castiglioni and Chiara Bertarelli**

Supporting Information

A conformationally driven mechanism in n-type doping of naphthalene diimide-bithiophene copolymer by 1H-benzimidazoles

Simone Cimò, Ilaria Denti, Lorenzo Rossi, Marco Cassinelli, Martina Rossi, Rossella Castagna, Garrett Lecroy, Alberto Salleo, Mario Caironi, Antonino Famulari, Chiara Castiglioni, Chiara Bertarelli*

Corresponding author: chiara.bertarelli@polimi.it

GIWAXS DATA

A summary of peak positions at different annealing temperatures and times is reported in the following tables:

Sample	In plane (Å)		Out Of Plane (Å)	
	(100)	(001)	(100)'	(010)
Pristine @70°C	26.07	13.84	25.44	3.88
Pristine not annealed	24.84	13.78	22.68	3.75
5 min @70°C	25.17	13.75	21.16	3.9
30 min @70°C	25.06	13.78	21.59	3.9
90 min @70°C	24.75	13.78	21.16	3.9

Table S1. Scattering peak position for the GIWAXS experiments at 70 [illegible] and pristine not annealed samples from the reduced 1D line profiles. The peaks are individuated along the horizontal (in plane, qxy) and approximately vertical (out of plane, ~qz) scattering directions.

Sample	In plane (Å)		Out Of Plane (Å)	
	(100)	(001)	(100)'	(010)
Pristine @110°C	26.07	13.84	25.44	3.88
Pristine not annealed	24.84	13.78	22.68	3.75
5 min @110°C	24.74	13.81	22.28	3.86
30 min @110°C	24.54	13.9	21.44	3.95
90 min @110°C	24.35	13.87	21.44	3.96

Table S2. Scattering peak position for the GIWAXS experiments at 110 ℃ and pristine not annealed samples from the reduced 1D line profiles. the peaks are individuated along the horizontal (in plane, qxy) and approximately vertical (out of plane, ~qz) scattering directions.

Coherence Length

Sample	In plane (Å)		Out Of Plane (Å)	
	(100)	(001)	(100)'	(010)
Pristine @70°C	209.06	210.65	48.33	17.07
Pristine not annealed	107.38	315.35	53.29	7.37
5 min @70°C	132.95	227.90	45.86	10.41
30 min @70°C	133.84	200.80	49.13	10.39
90 min @70°C	131.31	204.38	48.26	9.51

Table S3. Coherence length calculated from the peaks of the GIWAXS experiments at 70 ℃ and pristine not annealed samples. The coherence length was estimated from the Schererr equation for GIXS: $D = \frac{0.93*2\pi}{\Delta q}$

Sample	In plane (Å)		Out Of Plane (Å)	
	(100)	(001)	(100)'	(010)
Pristine @110°C	209.06	210.65	48.33	17.07
Pristine not annealed	107.38	315.35	53.29	7.37
5 min @110°C	128.94	279.85	59.07	8.34
30 min @110°C	125.39	259.59	59.46	8.27
90 min @110°C	120.71	284.49	67.16	7.38

Table S4.Coherence length calculated from the peaks of the GIWAXS experiments at 110 ℃ and pristine not annealed samples. The coherence length was estimated from the Schererr equation for GIXS: $D = \frac{0.93*2\pi}{\Delta q}$

GIWAXS texture analysis

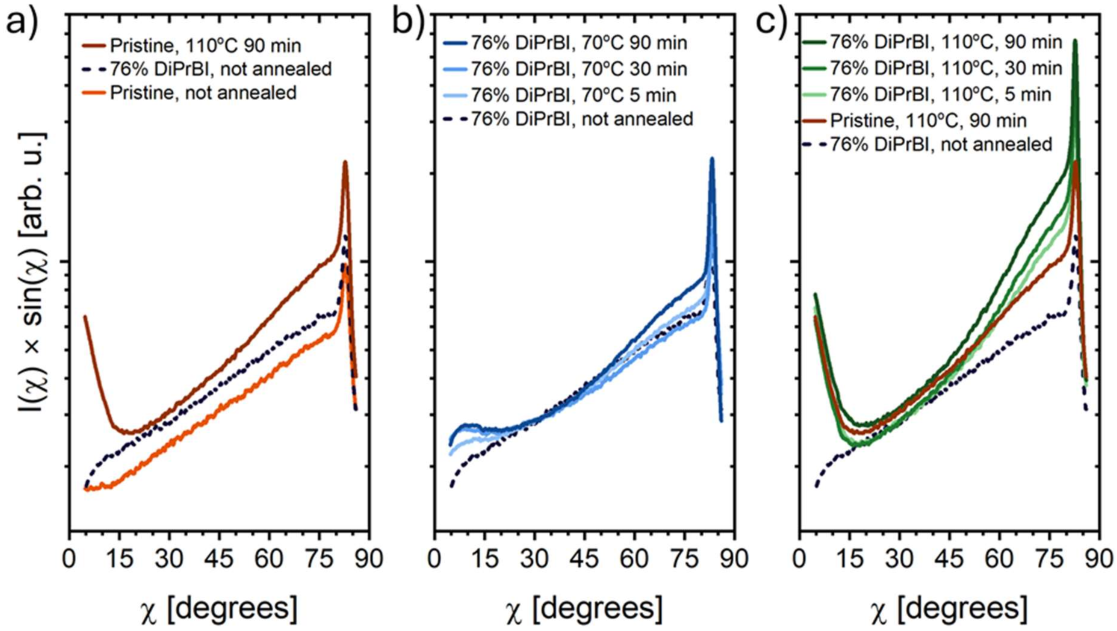

Figure S1: Lamellar (100)/(100)’ pole figures for P(NDI2OD-T2) with various annealing treatments for both pristine (i.e., undoped) and doped samples with 76% DiPrBI dopant. Data is shown as integrated intensity of the (100)/(100)’ peak weighted by the geometric correction of sin(χ), where χ is the polar angle relative to the substrate normal where χ = 0° represents out-of-plane scattering and χ=90° represents in-plane scattering. Note the log scale ordinate.

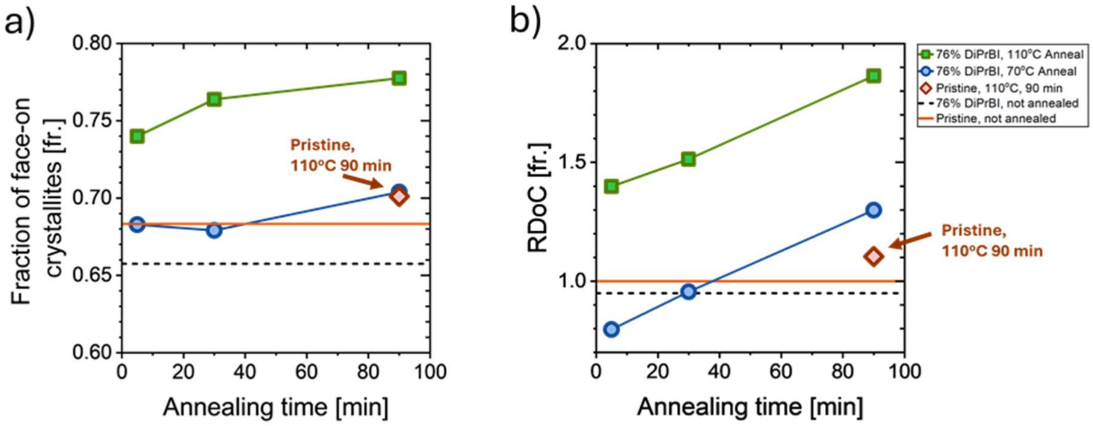

Figure S2: Summary of the **a)** relative amount of face-on crystallites and **b)** relative degree of crystallinity (RDoC) for P(NDI2OD-T2) with various annealing treatments for both pristine and doped samples with 76% DiPrBI dopant. The same legend applies to both figure panels. RDoC is referenced to the total integrated scattering intensity of the lamellar pole figure for the pristine sample with no annealing treatment (Figure S1.a). The fraction of face-on crystallites and RDoC is found from integrations of the lamellar pole figures shown in Figure **S1**. Annealing at 110°C generally increases the relative amount of face-on crystallites with increasing annealing time, alongside an increase in the RDoC with annealing time. Interestingly, the 110°C annealed samples with 76% DiPrBI dopant displayed the highest relative fraction of face-on crystallites and the highest RDoC for all annealing times when compared to the 70°C annealed samples with 76% DiPrBI dopant. The addition of 76% DiPrBI with no annealing initially causes a decrease in both the relative fraction of face-on crystallites and the RDoC.

Supplemental details on GIWAXS texture analysis

The total integrated scattering intensity (I_{total}) for the (100)/(100)' lamellar plane was found by integrating the lamellar pole figure shown in Figure S1 according to Equation 1.

$$I_{\text{total}} = \int_{\chi=0^\circ}^{\chi=90^\circ} I(\chi)\sin(\chi)d\chi \ (1) \tag{1}$$

Where $I\,(\chi)$ represents the scattering intensity of the (100)/(100)' lamellar plane as a function of polar angle χ.

The relative degree of crystallinity (RDoC) for various samples is defined relative to the (I_{total}) found for pristine sample with no annealing treatment according to Equation **2**.

$$\text{RDoC}^{\text{sample}} = \frac{I_{\text{total}}^{\text{sample}}}{I_{\text{total}}^{\text{pristine}}} \tag{2}$$

The relative fraction of face-on crystallites (fr_{face}) for a given sample is defined according to Equation **3.**

$$\text{fr}_{\text{face}} = \frac{\int_{\chi=45^\circ}^{\chi=90^\circ} I(\chi)\sin(\chi)d\chi}{\int_{\chi=0^\circ}^{\chi=90^\circ} I(\chi)\sin(\chi)d\chi} \tag{3}$$
